# Comparative Analysis of Pulsed and Continuous Wave Modes in High‐Intensity Laser Light Therapy: Implications for Deep Tissue Treatment

**DOI:** 10.1002/jbio.202400164

**Published:** 2025-05-19

**Authors:** Chironjeet Chaki, Luis De Taboada, Kwong Ming Tse

**Affiliations:** ^1^ Department of Mechanical and Product Design Engineering Swinburne University of Technology Melbourne Australia; ^2^ Enovis Lewisville Texas USA

**Keywords:** deep tissue treatment, laser therapy, light irradiance, pain management, photobiomodulation, pulse mode

## Abstract

High‐intensity laser at a wavelength of 1064 nm has gained significant attention in the field of therapeutic applications due to its potential to penetrate deeper. Continuous wave laser therapy, although effective, poses a challenge of elevated skin temperature. This study endeavors to explore the transition from continuous to pulsed laser, aiming to enhance light fluence in deep tissue while mitigating skin temperature rise. Investigating continuous versus pulsed wave in transdermal deep tissue light therapy, this research utilizes a high‐intensity laser at 1064 nm to optimize fluence in deep muscle tissue of the human knee, minimizing absorption‐driven skin temperature rise. Simulated parameters, including peak power (60 W), pulse width (2 ms), duty cycle (10%), frequency (50 Hz), as well as beam size of 20 mm, indicate that pulsed wave irradiation after 300 s achieved the lowest skin surface temperature (42.5°C) and the highest fluence (approximately 4.2 J/cm^2^).

## Graphical abstract

Pulsed laser irradiation enables enhanced deep tissue fluence with reduced skin temperature compared to continuous wave delivery. This study demonstrates that spreading energy over longer durations improves thermal safety while maintaining effective energy penetration for transdermal deep tissue light therapy.

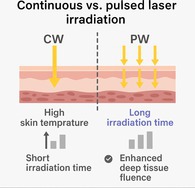

## 1 Introduction

High‐intensity laser therapy operating at a wavelength of 1064 nm has gathered considerable attention within the medical and therapeutic realms due to its potential to effectively treat deep tissues [1, 2]. This is primarily due to the efficient optical penetration of 1064 nm light, which exhibits relatively low absorption and scattering coefficients in biological tissues, enabling it to reach deeper muscle tissue compared to other wavelengths. While there is some consensus on the optimal light wavelengths and acceptable dosage ranges (irradiance and fluence), there remains a lack of agreement regarding the superiority of continuous wave (CW) or pulsed wave (PW) laser light, as well as the factors influencing the choice of pulse parameters [3, 4, 5, 6].

CW laser therapy presents a major challenge in the uncontrolled elevation of skin temperature during treatment, leading to patient discomfort, pain, and potential thermal damage. Conversely, pulsed light demonstrates distinct advantages, with evidence suggesting that it generates less tissue heating due to the presence of “quench periods” (times when the pulse is off) following pulse activation [1, 2, 6, 7]. This is especially beneficial in situations where more power is needed to convey light to deeper tissues in order to be certain that the target tissue receives enough energy [8]. Unlike laser ablation studies where ultrashort pulses are used within the thermal confinement regime to prevent heat diffusion, our study employs longer pulse durations (2–6 ms) that intentionally allow thermal diffusion. This approach helps regulate surface temperature, making it more suitable for nonablative, high‐intensity laser therapy applications targeting deep tissue. In this study, the application of HILT is specifically modeled for deep muscle tissue treatment in the human knee, based on an anatomically relevant geometry.

While elevated power levels in CW applications can result in surface tissue heating, pulsed light offers a potential solution. Although CW light at lower average power can theoretically deliver the same energy density over a longer time, this approach is often impractical due to extended treatment duration and the risk of cumulative thermal buildup. Pulsed light, by contrast, enables efficient energy delivery while mitigating surface heating through built‐in quench periods. A review by Hashmi et al. [6] summarized multiple studies on low‐level light therapy, highlighting the potential advantages of pulsed irradiation over CW delivery. In a study on anesthetized rats, Anders et al. [9] observed that pulsed light resulted in minimal thermal variation in brain tissue compared to CW exposure, while CW light at the same power density led to significant neurological abnormalities [6]. In addition, Ilicet et al. [10] reported that pulsed light did not harm tissues or the nervous system even at peak power densities of 750 mW/cm^2^ when it was directed for 120 s.

Furthermore, pulsed light not only offers enhanced safety, but could also hold the potential for greater effectiveness compared to CW light. Through the implementation of “quench periods” (pulse OFF times) to reduce tissue heating, pulsed light applications allow for the safe use of higher peak power densities, surpassing what is achievable with CW applications [10]. While doubling CW power density only marginally increases treatment depth and raises the risk of thermal damage [11], pulsed light at peak powers of ≥ 5 W/cm^2^ when appropriately pulsed, may produce little to no tissue heating. The use of pulsed light at higher peak powers helps reduce surface heating while enabling deeper light penetration, particularly, with high‐intensity 1064 nm lasers, as demonstrated in recent clinical and modeling studies [12, 13].

The potential therapeutic benefits of PW laser light over CW may, in part, be influenced by biological factors. Pulsed light sources commonly operate in the 2.5–10 000 Hz frequency range, with pulse durations of a few milliseconds [6, 14, 15, 16]. While the exact biological mechanisms remain under research, some studies suggest that pulsing parameters may interact more favorably with tissue responses compared to continuous exposure.

Short‐pulse lasers deliver high‐peak power in short durations, allowing the same amount of energy to be distributed over a longer period through pulsed exposure. Although energy deposition in tissue follows Lambert–Beer's law—with higher absorption near the surface—the key advantage of PW delivery lies in its ability to enable intermittent heat dissipation between pulses. By spreading energy over a longer irradiation time, pulsed delivery reduces cumulative heat buildup at the skin surface, thereby mitigating peak temperatures while still achieving effective fluence at deeper tissue layers. As demonstrated in our subsequent results, PW irradiation achieves lower skin temperatures compared to CW exposure under comparable energy delivery conditions. This supports the rationale for PW configurations in clinical applications where thermal safety is a critical consideration.

However, it is essential to note that the specific parameters of the laser treatment, including energy level and pulse duration, must be meticulously calibrated to ensure both safety and efficacy. Despite the growing prominence of high‐intensity laser therapy for deep tissue treatments, there remains a notable research gap regarding the optimization of treatment protocols, particularly, in simultaneously maximizing light irradiance at deep tissue levels, especially with high‐power lasers exceeding 30 W, while minimizing the risk of elevated skin surface temperatures.

Although previous research has explored the efficacy of different laser modes and their impact on tissue interaction, comprehensive studies addressing the intricate balance required for deep tissue treatments, emphasizing both efficacy and patient safety, are limited. Understanding how to harness the full potential of high‐power lasers for deep tissue therapy without compromising patient comfort and safety represents a critical research need in the field. Addressing this gap is vital for advancing the applicability and effectiveness of high‐intensity laser treatments, especially in clinical scenarios where deep tissue penetration is imperative.

This study investigates the emerging domain of high‐power pulsed laser therapy, where a computational approach, leveraging the finite element method and concurrent solutions of the radiative transfer equation (RTE) and bioheat transfer equation in COMSOL Multiphysics [17, 18, 19], is employed to maximize light irradiance on deep tissue while minimizing the risks associated with elevated skin temperature.

## 2 Materials and Methods

**FIGURE 1:**
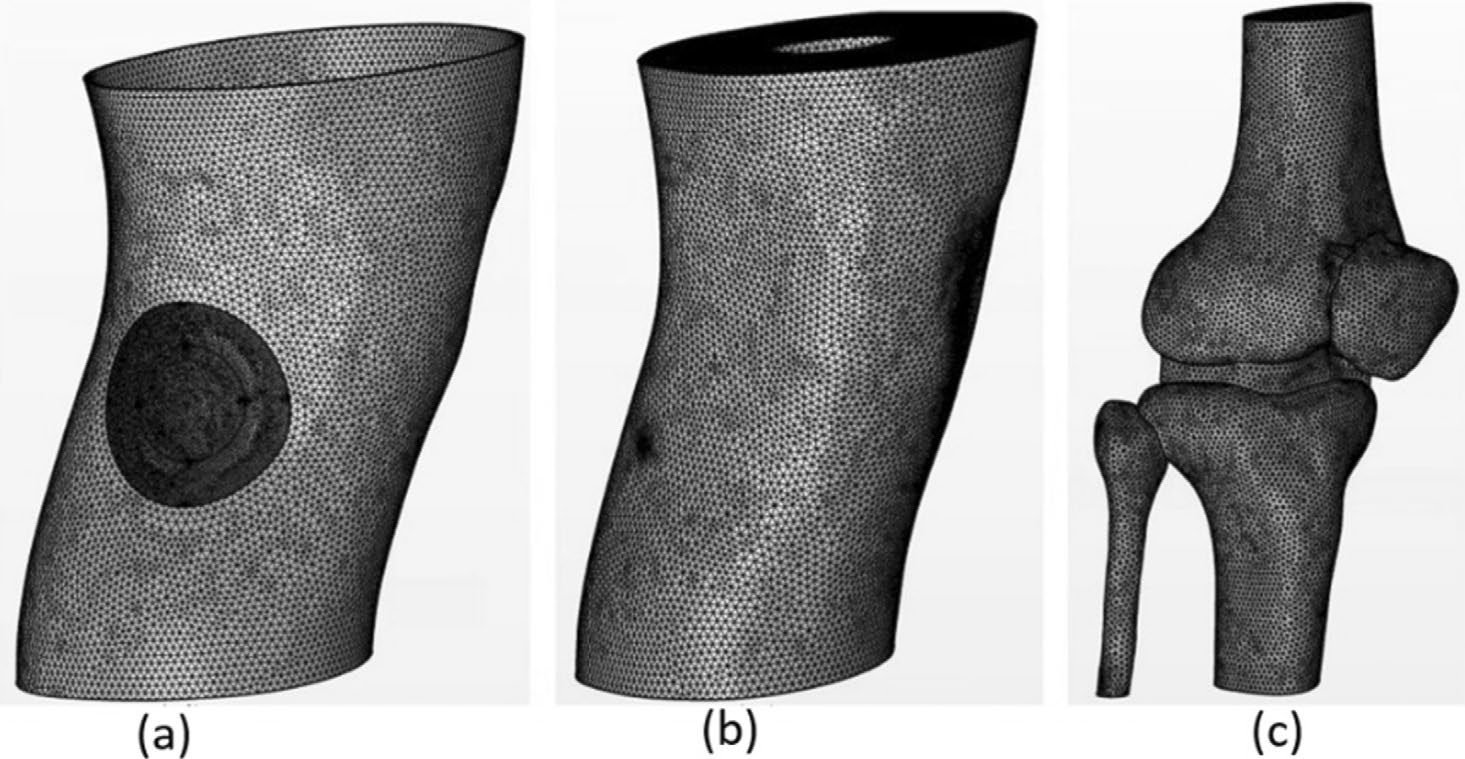
Anatomical human knee model—(a) Skin tissue layer (epidermis and dermis) with the round spot representing the irradiated region, (b) fat and muscle tissue layer, (c) bones and cartilage‐meniscus (combined into a single tissue) layer.

This investigation leverages a commercial finite element modeling software package to simulate the coupled phenomena of light propagation and heat transfer in the context of photobiomodulation applied to a human knee. Light propagation is simulated by solving the RTE in the form of diffusion approximation while heat transfer is solved through the bioheat equation. To incorporate the anatomically correct human knee model in our study, we built upon the detailed geometrical representation developed in our previous work [20]. Briefly, the model comprised 3D structures of various knee tissue layers, including skin (epidermis and dermis) (Figure 1a), fat, muscle (Figure 1b), bone, and a combined representation of cartilage and meniscus (Figure 1c). The “innermost muscle tissue” refers to the deepest section of the muscle layer located beneath the subcutaneous fat on the lateral side of the right knee, serving as the target zone for assessing fluence and temperature effects. This geometry was reconstructed from Visible Human Project data and meshed using COMSOL Multiphysics v5.6 software (details in [20]).

### 2.1 Propagation of Laser Light and Heat Transmission in Knee Tissue Layers

The study addresses the propagation of laser light in biological tissues through the utilization of the RTE formulation. Given the inherent intricacy in deriving a closed‐form exact solution for complex tissue geometries within the RTE, the commonly employed diffusion approximation is applied. In this present study, RTE was utilized in the form of a diffusion approximation, as described below in Equation (1), to evaluate the fluence distribution of skin‐applied irradiance within the tissues.

(1) $$\frac{1}{c}\frac{\mathrm{d\phi}\left(r,t\right)}{\mathrm{dt}}-\nabla D\left(r,t\right)\nabla \phi \left(r,t\right)+\mu_{a}\phi \left(r,t\right)=S\left(r,t\right)$$

where, $\phi \left(r,t\right)$ is the irradiance of laser light ($\mathrm{Wc}m^{-2})$, $D={\left[3\left(\mu_{a}+\mu_{s}^{\prime }\right)\right]}^{-1}$ is the coefficient of light diffusion (cm), $\mu_{a}$ is the coefficient of light absorption ($cm^{-1}$), $\mu_{s}^{\prime }$ is the coefficient of light's reduced scattering ($cm^{-1}$), c is the light speed in tissues ($ms^{-1}$), and S is the source of light ($\mathrm{Wc}m^{-3}$). With the subsequent equation, Dirichlet boundary condition was applied to model the source of light, $\left(1-G\right)\frac{P_{0}}{\pi r_{0}^{2}}$, where $P_{0}$ is the output power of laser (W), $r_{0}$ is the beam size (radius) of laser light (cm), and G is the reflectance between air and tissue [1].

Following the application of Equation (1) to ascertain the distribution of fluence within the tissue medium, Equation (2) was employed to numerically solve the time‐dependent bio‐heat transfer equation. This facilitated the computation of the thermal changes induced by light absorption in the tissue layers.

(2) $$\rho C_{p}\frac{\partial }{\partial t}T+\nabla .\left(-k\nabla T\right)=Q_{\text{light}}+Q_{\mathrm{bio}}$$

where, ρ is the density of different tissues (k g^−3^), $C_{p}$ is the heat capacity at constant pressure (J kg^−1^ K^−1^), *k* is the thermal conductivity of various tissues (Wm^−1^ K^−1^). Equations (3) and (4) were used to determine the heat source $Q_{\text{light}}\;\left(\mathrm{Wc}m^{-3}\right)$ resulting from the tissues' absorption of light and the heat transfer $Q_{\mathrm{bio}}\;\left(\mathrm{Wc}m^{-3}\right)$ resulting from the blood perfusion.

(3) $$Q_{\text{light}}=\mu_{a}\Phi$$

(4) $$Q_{\mathrm{bio}}=\rho_{b}C_{p.b}\omega_{b}\left(T-T_{b}\right)$$

where $\rho_{b}$ is the density of blood (kg^−3^), $C_{p.b}$ is the specific heat of blood $\left(J\;kg^{-1}K^{-1}\right)$, $\omega_{b}$ is the blood perfusion rate $\left(s^{-1}\right)$, and $T_{b}$ is the temperature of arterial blood (°C).

Table 1 provides an overview of the tissue optical and thermal characteristics that were gathered from published publications [21, 22, 23, 24] and utilized in this present study.

**TABLE 1:** Optical and thermal properties of various knee tissues in 1064 nm [21, 22, 23, 24].

	Properties	Unit	Skin		Subcutaneous Fat	Muscle
			Epidermis	Dermis		
Optical	Absorption coefficient (μ)	cm^−1^	0.21	0.5	0.6	0.26
	Reduced scattering coefficient ($\mu_{s}^{\prime }$)	cm^−1^	29.8	18	16.9	5
Thermal	Heat capacity at constant pressure ($C_{p}$)	J kg^−1^ K^−1^	3.39 × 10^3^		2.35 × 10^3^	3.42 × 10^3^
	Density (ρ)	K g^−3^	1.11 × 10^3^		9.11 × 10^2^	1.09 × 10^3^
	Thermal conductivity (k)	W m^−1^ K^−1^	3.7 × 10^−1^		2.1 × 10^−1^	4.9 × 10^−1^
	Arterial blood temperature ($T_{b}$)	^0^C	37		37	37
	Blood specific heat ($C_{p.b}$)	J kg^−1^ K^−1^	3.84 × 10^3^		3.84 × 10^3^	3.84 × 10^3^
	Mass density of blood ($\rho_{b}$)	k g^−3^	1.06 × 10^3^		1.06 × 10^3^	1.06 × 10^3^
	Blood perfusion rate ($\omega_{b}$)	s^−1^	2.57 × 10^−3^		2.3 × 10^−4^	2.7 × 10^−3^
	Metabolic rate	Wm^−3^	1 × 10^3^		1.801 × 10^2^	1 × 10^3^

In order to examine how light fluence (J/cm^2^) and tissue temperature (°C) affect knee tissues in an anatomical human knee model, this study simulated the application of commonly utilized CW and PW dosing parameters for HILT directly on the lateral side of the right knee. While we did not include a side‐by‐side simulation at a fixed average power (e.g., 6 W), the set of PW scenarios explored already spans a wide range of average powers that overlap with CW conditions. The observed trends—particularly, reduced peak skin temperatures in pulsed modes—reflect the benefits of temporal energy modulation, which align with our study's objective without requiring fixed‐average‐power matching. Table 2 provides specifics on the parameters and their values.

**TABLE 2:** Laser parameters and values used for simulation.

Laser parameters	Values
Wavelength (nm)	1064
Laser mode	CW, PW
CW power output (W)	30
PW peak power (*P*_peak_) (W)	30, 40, 50, 60
PW average power (*P*_avg_) (W)	3, 4, 5, 6, 9, 12, 15, 18
Beam size (mm)	20
Pulse frequency (Hz)	50
Pulse width (ms)	2, 3, 4, 5, 6
Duty cycle (%)	10, 15, 20, 25, 30
Irradiation time (s)	60–300

## 3 Results

### 3.1 Effect of Peak Power on Tissue Temperature: CW Versus PW

**FIGURE 2:**
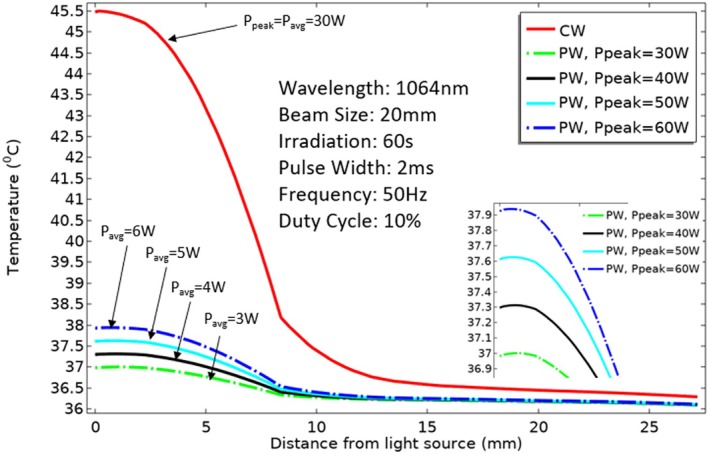
Effect of peak power on knee tissue layers.

In this study, a simulation was conducted to make a comparative analysis of the impact of laser pulses and CW delivery techniques on skin temperature. The simulation utilized a wavelength of 1064 nm, a beam radius measuring 20 mm, a peak power output of 30 W, a pulse duration of 2 ms, a duty cycle of 10%, and an overall irradiation duration lasting 60 s. The results of the simulation revealed a remarkable temperature reduction on the skin surface when using the laser pulse delivery mode as opposed to CW delivery mode, which is shown in Figure 2. After 60 s of irradiation, the skin surface temperature of the pulsed laser with *P*_peak_ = 60 W, 10% duty cycle (*P*_avg_ = 6 W) and pulse width of 2 ms reached approximately 38°C. In contrast, the skin temperature reached around 46°C under CW conditions, where *P*_peak_ = *P*_avg_ = 30 W. This significant difference in temperature highlights the superior thermal regulation achieved with pulse irradiation. Figure 2 also shows that while the peak power of PW increased by 10 W (*P*_avg_ = 1 W), the skin's surface temperature rose by 0.3°C. One notable advantage associated with employing pulsed laser delivery was the successful reduction of laser power, resulting in diminished temperature fluctuations within each individual tissue layer. By employing pulsed‐wave radiation with a 10% duty cycle, the peak power of 60 W was effectively reduced to an average power of 6 W. This substantial reduction in laser power resulted in a considerable decrease in the amount of heat transferred to the tissues.

### 3.2 Comparing Fluence Distribution in Deep Tissue: CW Versus Pulsed Laser Power

**FIGURE 3:**
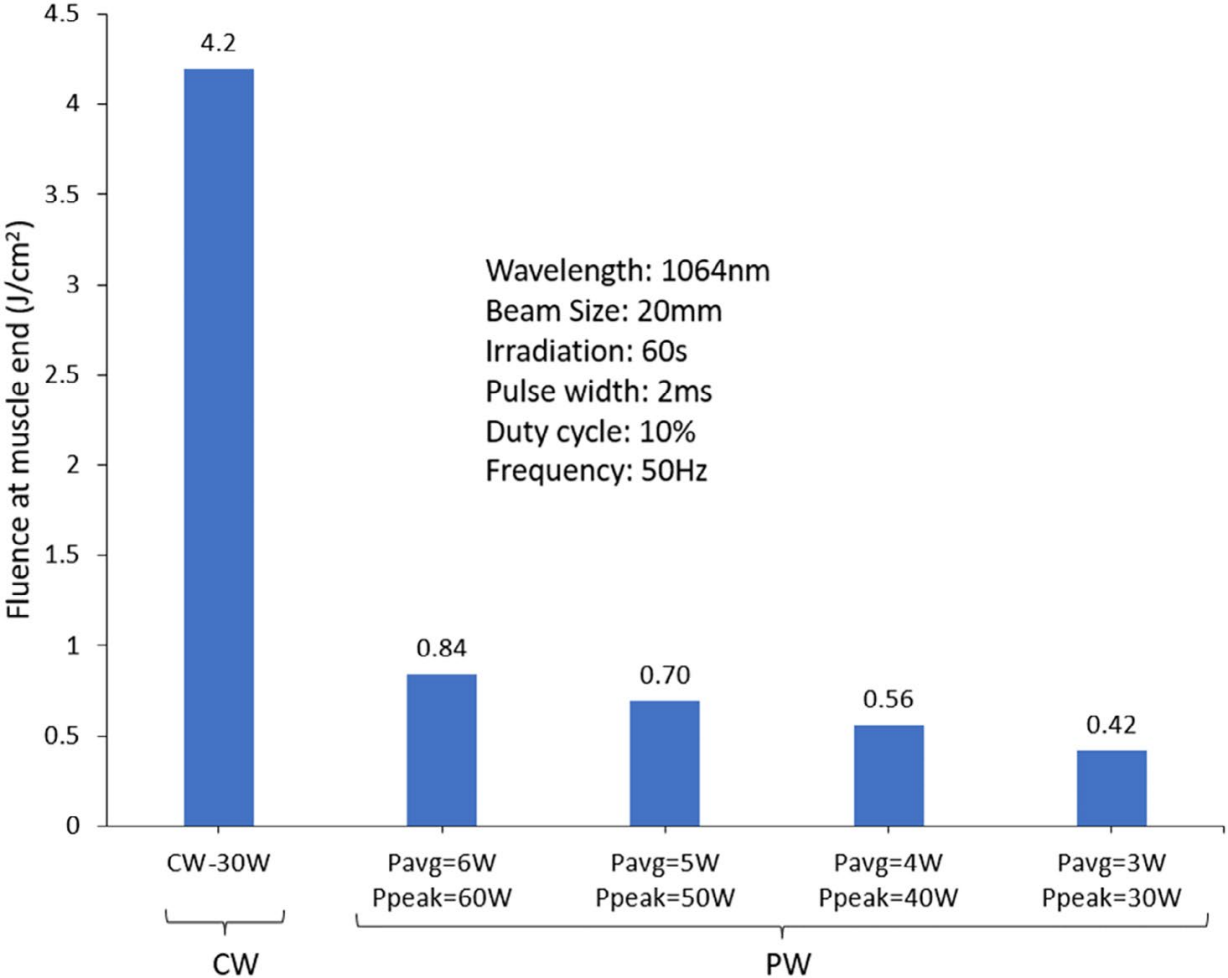
CW–PW comparison of fluence distribution measured at the deepest muscle layer (innermost muscle tissue) in the lateral side of the human knee model. The graph illustrates that fluence scales with average power under identical irradiation time and beam parameters.

The utilization of laser pulses offers a unique advantage in reducing the temperature at the skin surface, as shown in Figure 2; however, it is worth noting that this reduction in temperature also correlates with a decrease in energy density within the deeper tissue layers. Figure 3 illustrates that in the same above laser configuration (wavelength = 1064 nm, beam radius = 20 mm, pulse width = 2 ms, frequency = 50 Hz, duty cycle = 10%, and irradiation time = 60 s) CW (*P*_peak_ = *P*_avg_ = 30 W) produces a fluence of 4.2 J/cm^2^ in the deep muscle tissue. On the other hand, the pulsed laser with *P*_peak_ = 60 W (10% duty cycle, *P*_avg_ = 6 W) shows a fluence of 0.84 J/cm^2^, which is notably lower than its CW counterpart. As shown in Figure 3, fluence values decreased proportionally while peak power reduced progressively. Figure 3 also shows that *P*_peak_ = 30 W (*P*_avg_ = 3 W) yields a fluence of 0.42 J/cm^2^, vividly illustrating the effect. Furthermore, by further reducing laser power to *P*_avg_ = 3 W and *P*_peak_ = 30 W, a fluence of 0.42 J/cm^2^ is achieved. As expected, fluence deposition at the innermost muscle tissue (refers to the deepest part of the muscle layer beneath subcutaneous fat on the lateral side of the knee) scales proportionally with the average power when other parameters such as beam size, wavelength, and irradiation time are held constant.

While this study did not include a direct comparison between CW and PW modes at the same average power (e.g., 6 W), the selected irradiation settings were chosen to reflect practical clinical conditions. In such scenarios, continuous high‐power CW delivery is often limited due to thermal safety concerns. The presented PW configurations demonstrate that high‐peak power delivery with intermittent energy application can achieve acceptable deep tissue fluence while maintaining lower surface temperatures.

### 3.3 Effect of Peak Power and Laser Irradiation Period in Skin Temperature

**FIGURE 4:**
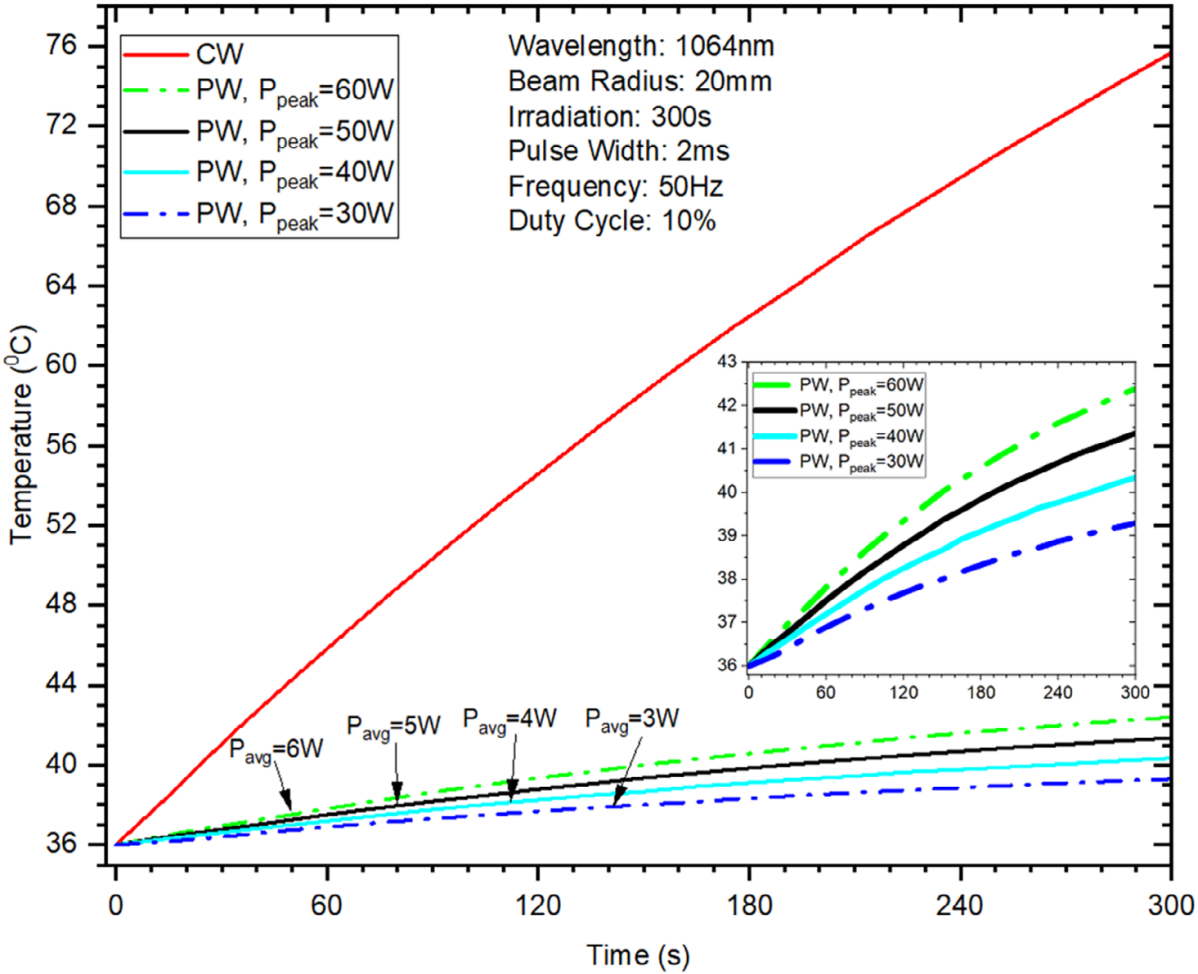
Effect of laser irradiation (from 60 to 300 s) on skin surface temperature.

In pulsed laser irradiation, extending the irradiation duration enables the delivery of higher fluence to deep tissues, thus offsetting the decrease in applied irradiance to the skin. The results are shown in Figure 4, which illustrates how pulsed laser irradiation achieves a useful balance in skin surface temperature reduction compared to CW. Figure 4 shows that after 300 s of irradiation, the skin's surface temperature rose by about 2°C (to 39°C) in PW at *P*_peak_ = 30 W (*P*_avg_ = 3 W), however, for CW (*P*_peak_ = *P*_avg_ = 30 W) it reached to approximately 76°C (skin temperature increased by 40°C which beyond the acceptable temperature limit for skin tissue in accordance with the international electrotechnical commission's (IEC) safety guidelines. Figure 4 also shows that if the peak power increased from 30 to 60 W (*P*_avg_ = 6 W) with the application of PW, the skin's surface temperature rose by about 5.5°C (42.5°C, a temperature window suitable for skin without exceeding its tolerance threshold [25]).

The temperature evolution was assessed using a fixed 300‐s irradiation period for all pulsed laser cases as this approach reflects typical clinical constraints, where treatment time is often limited by patient comfort and workflow efficiency. Although different average powers were applied, the focus was on evaluating thermal behavior under a consistent and practical treatment duration rather than delivering equal energy in all cases.

### 3.4 Effect of Duty Cycle and Repetition Rate

**FIGURE 5:**
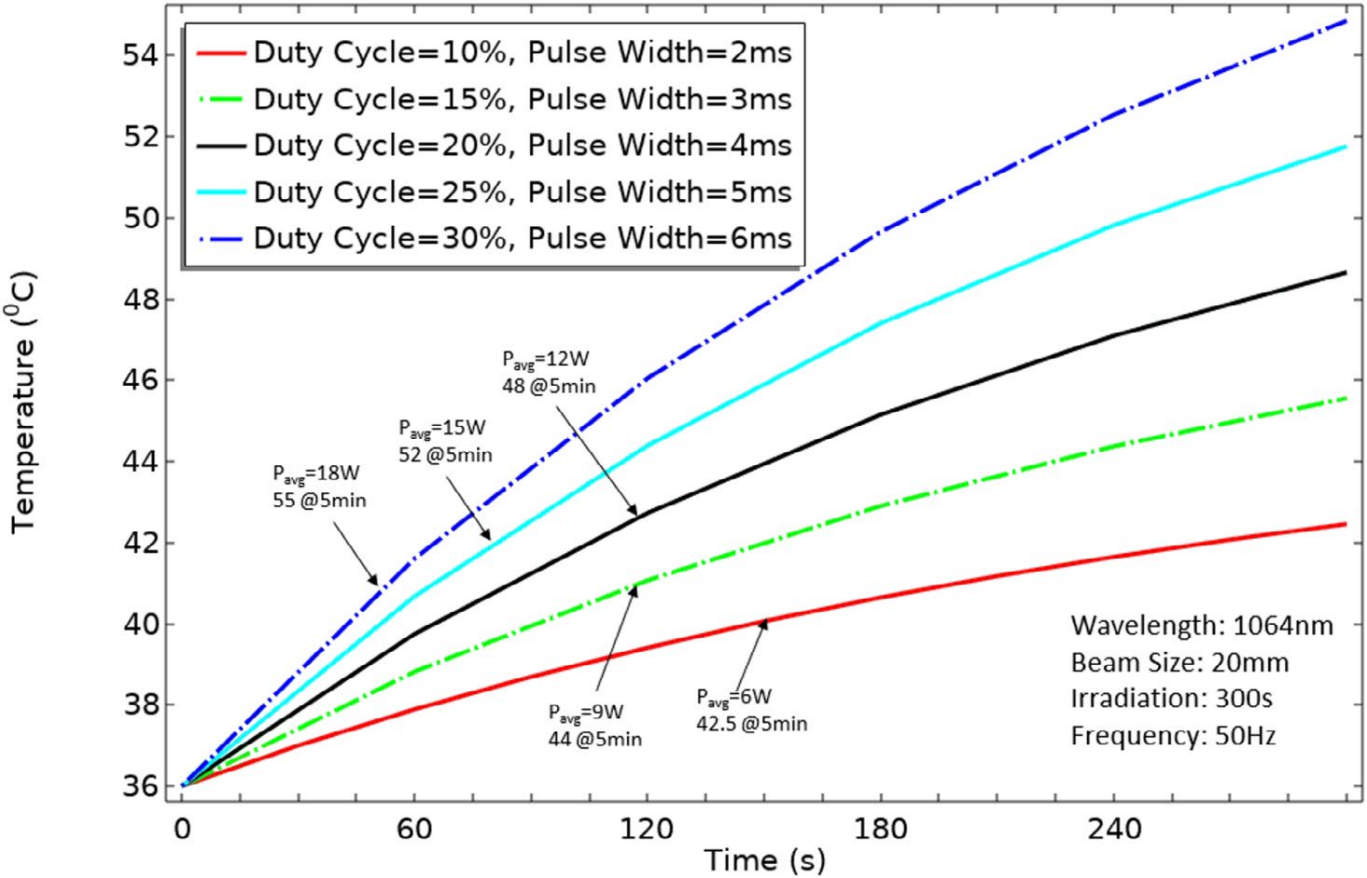
Effect of duty cycle on skin temperature.

**FIGURE 6:**
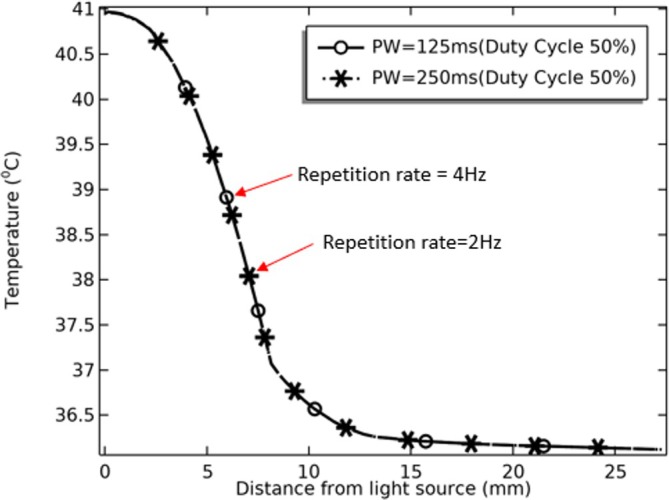
Effect of frequency on skin temperature.

Maintaining the right duty cycle is essential for managing the temperature of the skin's surface. To investigate the phenomenon, a transient simulation was conducted varying the duty cycle at wavelengths of 1064 nm, with a beam radius of 20 mm. Five different duty cycles (10% [*P*_avg_ = 6 W], 15% [P_avg_ = 9 W], 20% [P_avg_ = 12 W], 35% [P_avg_ = 15 W] 30% [P_avg_ = 18 W]) with a constant peak power of 60 W were examined for a total of 300 s of pulsed laser irradiation (2–6 ms) to observe their impact on thermal changes at the skin surface. The results are depicted in the following Figure 5, as the duty cycle increases, a corresponding gain in temperature was observed on the skin surface. For instance, when the duty cycle was set at 10% (with a pulse width of 2 ms and an average laser power of 6 W), the skin surface temperature reached approximately 42.5°C. On the other hand, as the duty cycle increased to 15%, 20%, 25%, and 30%, the temperature rose to roughly 44°C, 48°C, 52°C, and 55°C, respectively. However, the repetition rate plays a unique role, such as the acoustic effect on blood vessels induced by rapid laser pulsing leading to improved tissue perfusion and potential therapeutic benefits in certain applications; it is evident that changes in the repetition rate do not impact temperature on the skin surface when the duty cycle remains constant, as depicted in Figure 6 which shows that altering the repetition rate from 4 to 2 Hz (with a pulse width of 125–250 ms) while maintaining a consistent duty cycle of 50% does not lead to any significant thermal changes at the skin surface. The constancy of the duty cycle ensures that the laser power remains unchanged, resulting in the stability of tissue temperature. Consequently, fluctuations in the repetition rate do not cause variations in the thermal impact on the skin.

It is worth noting that in this study, the duty cycle was varied while maintaining a fixed peak power of 60 W, which naturally resulted in varying average power. This setup was intended to reflect practical HILT applications, where laser systems often operate with fixed peak outputs.

### 3.5 Skin Applied Irradiance for Optimal Fluence in Deep Tissue and Skin Temperature

**FIGURE 7:**
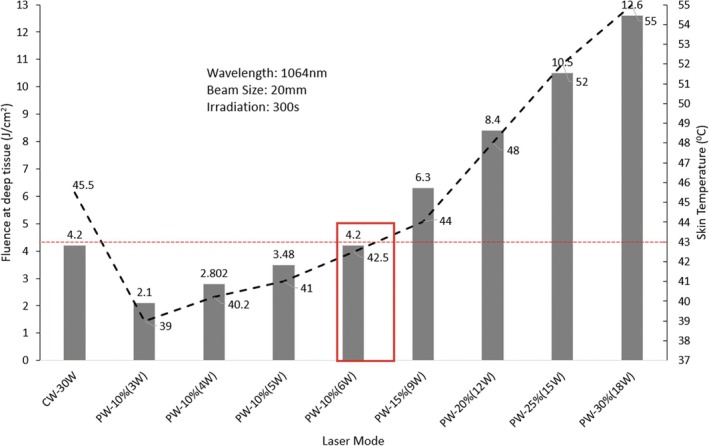
Optimal pulsed laser parameter setting for maximizing fluence in deep tissue and minimizing temperature change on skin surface.

In this section, we investigated the association among laser power, fluence, and skin temperature to determine the maximization of fluence in deep tissue (innermost muscle tissue) and the minimization of temperature rise in skin surface (reasonable thermal sensation which skin can tolerate). In Figure 7, a CW laser operating at 30 W was tested with a 60 s of irradiation, while a PW laser at peak power of 60 W (various average power levels (3–6, 9, 12, 15, and 18 W) was examined with a 300 s irradiation.

The results showed that a PW laser with peak power of 60 W (*P*_avg_ = 6 W, 10% duty cycle) produced the optimum balance between maximizing fluence and minimizing skin temperature. This laser power produced a fluence of 4.2 J/cm^2^ and a temperature of 42.5°C, which is below the commonly referenced safety threshold of 43°C for skin surface temperature based on IEC 60601‐2‐22 laser safety standards. Our findings suggest that a PW laser power of 6 W is a safe and effective way to deliver laser treatment. More investigation is required to measure the long‐term efficacy of this laser treatment approach across various skin conditions.

CW laser operating at 30 W was tested with a 60 s irradiation period, while PW lasers at various power levels (3–6, 9, 12, 15, and 18 W) were examined with a 300 s irradiation period. The objective was to compare these two laser modes and identify optimal settings that maximize fluence while ensuring skin temperature remains below the tolerable threshold of 43°C. Our analysis of light transmission and thermal effects, as illustrated in Figure 7, suggests that within the context of 1064 nm wavelength, the selected pulsed laser parameters achieved favorable outcomes in maximizing deep tissue fluence while minimizing skin surface temperature. It demonstrated the maximization of light fluence into deep muscle tissue while simultaneously minimizing skin temperature increases. It is important to note that the CW case was simulated for 60 s, while all PW scenarios used 300 s of irradiation. This was a deliberate design choice, as high‐power CW exposure beyond 60 s exceeded thermal safety limits. Pulsed modes, due to their intermittent delivery, allow for longer irradiation while maintaining tolerable skin temperatures.

## 4 Discussion and Conclusion

The simulated results highlight the potential advantages of PW laser therapy for transdermal deep tissue light therapy (tDTLT), particularly, in reducing surface heating while enabling adequate fluence delivery to deep muscle tissues in an anatomically correct human knee model. While our findings support PW as a safer alternative to CW in the tested configurations, we acknowledge that no direct CW–PW comparison was performed at identical average power levels, which limits the strength of these conclusions. Our comprehensive simulation framework incorporating Peak Power (*P*_peak_), Pulse Width, Duty Cycle, Frequency, and Beam Size provides valuable data and insights for advancing laser therapy applications in medicine and therapeutics. PW delivery achieved a significantly lower skin surface temperature (42.5°C after 300 s of irradiation) while simultaneously delivering the notable fluence (approximately 4.2 J/cm^2^) to the innermost muscle tissue. This indicates that the PW delivery has the capability to be a safe and effective alternative to the traditional CW mode, offering improved deep tissue penetration with reduced thermal risk. Further comparative analysis confirms the superiority of PW delivery. Specifically, a PW configuration with *P*_peak_ = 60 W, 10% duty cycle (*P*_avg_ = 6 W), and a pulse width of 2 ms demonstrably outperforms CW conditions (*P*_peak_ = *P*_avg_ = 30 W) in terms of thermal management. While the CW treatment reached a skin temperature of approximately 46°C after only 60 s, the PW procedure accomplished a substantially lower temperature (42.5°C) even after a longer irradiation time (300 s). This substantial temperature reduction with PW is clinically relevant, as elevated skin temperatures during laser therapy can pose various risks, including epidermal damage and pain.

The advantage of PW delivery lies in its ability to effectively reduce peak power (*P*_peak_) while maintaining the average power (*P*_avg_). This translates to a significant decrease in heat transfer to tissues. The current study illustrates this concept by employing a 10% duty cycle with *P*_peak_ = 60 W, corresponding to *P*_avg_ = 6 W. This aligns with the critical need to limit thermal impact on tissues, indicating that pulsed laser therapy has the potential to provide improved safety and effectiveness in tDTLT applications. Further exploration of different duty cycles reveals their role in regulating skin surface temperature. As the duty cycle increases, a corresponding rise in skin temperature is observed. This emphasizes the need for careful consideration of duty cycle settings based on the desired balance between deep tissue fluence and skin surface temperature control. Interestingly, the repetition rate, while having its unique role, does not significantly impact skin temperature when the duty cycle remains constant. This stability in tissue temperature showcases the potential benefits of pulsed laser therapy, providing a level of predictability and control necessary for safe and effective treatment.

Clinical studies consistently highlight challenges associated with CW laser therapy, particularly, the risk of elevated skin temperatures. Our study's exploration of CW laser therapy as an alternative aligns seamlessly with the clinical imperative to develop strategies that mitigate these thermal challenges. It is paramount to acknowledge the limitations of our current numerical study. While the simulation provides valuable insights, it is unable to fully replicate the complexity of biological tissues and dynamic responses observed in clinical scenarios. Our study employed a simplified tissue model, assuming homogeneity and uniformity, which might oversimplify real‐world intricacies of tissue behavior. Moreover, the lack of biological variability, such as diverse skin types, conditions, and patient responses, limits the generalizability of the results. Clinical trials encompassing a diverse patient population are essential for comprehensive validation and to ensure the proposed PW laser therapy is applicable across various clinical contexts. Additionally, our study primarily focuses on short‐term outcomes during the simulation period. The long‐term effects and sustainability of the proposed PW laser therapy regimen remain unexplored. Future research should investigate the durability and longevity of treatment effects, providing a more comprehensive understanding of the therapy's efficacy over extended periods.

While this study references temperature thresholds (e.g., 43°C for skin) based on established safety standards, we acknowledge that thermal damage is a function of both temperature and exposure duration. The Arrhenius damage integral provides a more comprehensive framework for evaluating heat‐induced tissue injury and could enhance the accuracy of future simulations, particularly, under extended exposures. Although not included in the current model, we propose its integration in future studies to better predict safety margins. Additionally, we clarify that the goal of tDTLT, as pursued in this study, is to achieve effective fluence deposition within deep muscle tissues while maintaining surface temperatures below safety thresholds, ensuring both therapeutic efficacy and patient comfort within clinically practical irradiation times. Beyond these considerations, the current study did not include matched average power comparisons between CW and PW modes or simulations with equal energy deposition by adjusting irradiation time. Such analyses could more precisely isolate the influence of pulsing and energy delivery efficiency. Similarly, the duty cycle was examined with a fixed peak power, leading to changes in average power; future studies could explore the inverse—holding average power constant while varying peak power to better understand duty cycle‐specific effects. These directions offer promising opportunities for future refinement of simulation‐based laser therapy evaluation.

In conclusion, our study establishes potential strategies for optimizing laser therapy parameters through the exploration of PW delivery at 1064 nm for tDTLT. While promising outcomes require further investigation through clinical trials and long‐term studies, this work lays a robust theoretical foundation for future experimental and clinical investigations in diverse clinical settings, and we propose future models incorporate Arrhenius‐based thermal damage criteria to further improve treatment safety evaluation.

## Conflicts of Interest

The authors declare no conflicts of interest.

## Data Availability

The data that support the findings of this study are available from the corresponding author upon reasonable request.
